# Male Seminal Fluid Substances Affect Sperm Competition Success and Female Reproductive Behavior in a Seed Beetle

**DOI:** 10.1371/journal.pone.0123770

**Published:** 2015-04-20

**Authors:** Takashi Yamane, Julieta Goenaga, Johanna Liljestrand Rönn, Göran Arnqvist

**Affiliations:** 1 Noguchi 350–17, Kakogawa, Japan; 2 Aarhus Institute of Advanced Studies, Aarhus University, Aarhus, Denmark; 3 Animal Ecology, Department of Ecology and Genetics, Uppsala University, Uppsala, Sweden; CNRS, FRANCE

## Abstract

Male seminal fluid proteins are known to affect female reproductive behavior and physiology by reducing mating receptivity and by increasing egg production rates. Such substances are also though to increase the competitive fertilization success of males, but the empirical foundation for this tenet is restricted. Here, we examined the effects of injections of size-fractioned protein extracts from male reproductive organs on both male competitive fertilization success (i.e., P2 in double mating experiments) and female reproduction in the seed beetle *Callosobruchus maculatus*. We found that extracts of male seminal vesicles and ejaculatory ducts increased competitive fertilization success when males mated with females 1 day after the females’ initial mating, while extracts from accessory glands and testes increased competitive fertilization success when males mated with females 2 days after the females’ initial mating. Moreover, different size fractions of seminal fluid proteins had distinct and partly antagonistic effects on male competitive fertilization success. Collectively, our experiments show that several different seminal fluid proteins, deriving from different parts in the male reproductive tract and of different molecular weight, affect male competitive fertilization success in *C*. *maculatus*. Our results highlight the diverse effects of seminal fluid proteins and show that the function of such proteins can be contingent upon female mating status. We also document effects of different size fractions on female mating receptivity and egg laying rates, which can serve as a basis for future efforts to identify the molecular identity of seminal fluid proteins and their function in this model species.

## Introduction

Multiple mating by females occurs in most animal taxa [1–4]. Irrespective of whether and how females benefit from polyandry, it results in sperm competition among males [5,6]. Males have evolved suites of morphological, behavioral and physiological traits that represents sperm competition adaptations which, in various ways, increase the probability that a given male will fertilize the eggs of his mate [7–9]. In insects as well as in most other taxa with internal fertilization, males transfer seminal fluid proteins to females during mating. These ejaculate proteins have profound effects on female physiology and behavior [10,11]. The best documented effects include (i) elevation of egg production and egg laying rates and (ii) inhibition of future remating, both of which benefits the donor male in terms of sperm competition. The diverse effects of seminal fluid proteins have been assessed in many different insect taxa, but none are as well characterized as the accessory gland proteins of *Drosophila melanogaster* [12–15].

Male seminal fluid proteins are often thought to also confer a male fitness benefit through increased male competitive fertilization success. Such effects can be mediated by a direct influence on sperm which increases their viability or motility [16–18]. However, it has also been suggested that seminal fluid proteins can have hormonal effects in females that elevate male competitive fertilization success by, for example, increasing the rate the rate of sperm uptake/retention by females, but evidence for such effects is currently limited to *D*. *melanogaster* [19–24].

Although male seminal fluid substances are in most taxa primarily produced by male accessory reproductive glands, such substances can also be produced by reproductive organs such as the testes or ejaculatory ducts [25–28]. Because research in this field is often restricted to studies of the accessory glands, information on the reproductive effects of substances from organs other than the accessory glands from a range of species would further our understanding of the diversity of male seminal fluids proteins among species.

Seed beetles of the genus *Callosobruchus* have recently emerged as a model system for studies of sexual selection, sperm competition and sexual conflict (e.g., [29–36]). Briefly, females in this genus mate multiply and males transfer large ejaculates (up to 8% of their body weight) which contain a large number of sperm cells (more than 50000) as well as a complex cocktail of seminal fluid substances. The male genitalia of several species bear sclerotized spines that puncture the female reproductive tract, causing injury in females, and females have evolved morphological counter-adaptations in their reproductive tract which apparently alleviate the harm caused by males. Hotzy et al. [37] recently showed that male genital spines were associated with increased rate of diffusion of male seminal fluid substances from the female reproductive tract into the female body cavity. Genital spines were also correlated with elevated competitive fertilization success in males, suggesting that spines may in part benefit males by increasing the efficacy of seminal fluid proteins. Here, we directly test the hypothesis that some seminal fluid proteins act to increase male competitive fertilization success by artificial injections of male reproductive organ—derived substances. This technique has been used in a large number of insect studies to examine the effects of male seminal fluid substances on female physiology and behavior (e.g., [38–44]). In the present study, we used artificial injection to assess the effects of different male reproductive organ-derived substances on second male sperm precedence as well as on female reproductive behavior in *C*. *maculatus*. To further characterize these effects, we also examined the effects of different molecular-weight fractions of accessory gland extracts on these responses.

## Methods

We used the SI4 strain of *C*. *maculatus*, which is a well-adapted strain that derives from a population originally collected in South India in 1979 and attained from P. Credland (University of London). The stock culture was maintained in jars containing mung beans, *Vigna radiata*, in a chamber under controlled conditions (temperature, 29°C; relative humidity, 60%; 12-h light/12-h dark cycle) at Uppsala University. We first obtained virgin beetles by individually isolating mung beans, on which mated females of the stock culture had laid eggs, in separate wells in 24-well tissue culture plates. To avoid crowding, four to five virgin beetles of each sex were introduced to Petri dishes (height, 1.5 cm; diameter, 9.0 cm) containing 150 to 200 mung beans and were allowed to mate and oviposit for 1 day, to minimize downstream parental age effects. Beans on which these females had laid eggs (1–3 eggs per bean) were then transferred to a 48-well tissue culture plate (1 bean per well) and virgin individuals (of ≤2 days adult age) that emerged from those eggs were then used in the experiments described below.

### Ethics Statement

Formal ethical approval was not required for the research reported here, as insects were used as the model organism. Efforts were nevertheless made to minimize potential suffering: beetles were reared on their natural host under conditions mimicking natural conditions and all injections were made under CO_2_ anesthesia.

### Preparation of male reproductive organ—derived extracts and fractions of accessory gland extract

At 0 to 3 days after emergence, approximately 200 adult virgin males were dissected in a drop of Milli-Q water on a silicone medium under a binocular microscope. We then performed two sets of preparations. First, to produce extracts of organ-specific male substances, the accessory glands, testes, and seminal vesicle with ejaculatory duct attached were removed separately with forceps and micro-scissors. Organ excision was performed very carefully, to avoid potential cross-contamination, and any samples were organs ruptured were discarded. Ten to twenty-five of each of the excised organs were stored in 10 μL Milli-Q water in Eppendorf tubes (1.5 mL) at −80°C until use. The frozen water containing the organs was transferred to a 15-mL glass test tube, homogenized with a glass rod in 1000 μL Milli-Q water, and centrifuged (6000 rpm, 10 min, 4°C). The supernatant was removed and the pellets were re-extracted twice with 500 μL Milli-Q. The supernatants were pooled (to minimize within-group varation), completely lyophilized and stored at −80°C.

Second, to produce distinct size-fractions of accessory gland extract, stored accessory glands were homogenized in Milli-Q water (100 glands per 500 μL) and centrifuged (6000 rpm, 10 min, 4°C). The supernatant was removed and the pellet was re-extracted twice with 250 μL Milli-Q water per 100 glands and the supernatants were pooled. The aqueous accessory gland extract was then dialyzed at 4°C against a 100-fold excess of Milli-Q water by using a dialysis bag that retained compounds with a molecular mass ≥25 kDa (Spectra/Por 7; Spectrum laboratories Inc., California, USA). The external solution (containing compounds with a molecular weight <25 kDa) was completely lyophilized and stored at −80°C as the low molecular weight fraction (Fraction I). The procedure was repeated with a dialysis bag (Spectra/Por 7) that retained compounds ≥50 kDa. The external solution (containing compounds with a molecular weight of 25–50 kDa) and dialysate (≥ 50 kDa) were completely lyophilized and kept at −80°C as the middle molecular-weight fraction (Fraction II) and the high molecular-weight fraction (Fraction III), respectively.

### Injection of male reproductive organ—derived extracts

To assess the phenotypic effects of male-derived substances, females (for ages and sample sizes, see below) were injected with such substances. Females were first placed in a plastic tube and lightly anesthetized with CO_2_ and then fixed with glue to a stand. Male reproductive organ—derived extracts were dissolved in Milli-Q water to the indicated concentration and volume (see below) and injected into the female abdomen (between the 2^nd^ and the 4^th^ sternite) by means of a fine glass capillary tube connected to an oil pressure micro-injector (CellTram Oil; Eppendorf AG, Hamburg, Germany) supported by a micro-manipulator. Control females received injections of the same volumes. Injections were conducted in the laboratory at 25°C.

### Control treatment

We used a solvent-only negative sham control, where control females were injected with the solvent (i.e., Milli-Q water) without male seminal substance extract. This simultaneously controls for any effects of injection per se as well as any effects of the solvent. We note here that (i) injection of male derived substances did not significantly decrease female lifespan relative to the control (see below) and (ii) the different treatment levels also serve as internal controls relative to one another, in the sense that all involved the injection of male derived proteins into females.

### Effects of male reproductive organ—derived extracts on sperm competitive ability

To assess whether seminal fluid compounds affects male competitive fertilization success, we used a standard sterile male technique [8] in which females are mated to two males in succession one of which is sterilized and one of which is not. Virgin males (1–3 days old) were sterilized by exposures to gamma radiation using a caesium source at the Rudbeck laboratory at Uppsala University (dose, 80–90 Gy). This dose causes life-long sterility in male seed beetles [45], by inducing chromosomal mutations in sperm that cause early embryonic death in offspring [8], but does not notably impair male competitive fertilization ability [46]. One virgin female (0–2 days old) was placed with one sterilized virgin male in a small plastic Petri dish (height, 1.0 cm; diameter, 3.0 cm), at 25°C, and the couple was observed continuously. Following one complete mating, the male was immediately removed upon disengaging from the female to prevent repeated mating. The mated females were then kept individually in Petri dishes (height, 1.5 cm; diameter, 6.0 cm) containing 10 mung beans for either 1 or 2 days, to allow assessment of whether effects were contingent upon inter-mating interval, and then re-mated with a non-sterilized virgin male (1–3 days old) using the same protocol as for the first mating. When re-mating did not occur after 60 min, the process was repeated with second virgin male and females that did not re-mate upon their second opportunity were discarded. Re-mated females were injected immediately after their second mating either with one of the male reproductive organ-derived extracts (injection volume, 0.05 μL; concentration, 0.25 mg/μL) or with one of the accessory gland size-fractions (injection volume, 0.05 μL, concentration 0.5 mg/μL). After injection, females were transferred to a Petri dish (height, 1.5 cm; diameter, 9.0 cm) containing 100 mung beans and kept there until death. We then (8 to 20 days after the death of each female) counted all hatched and unhatched eggs produced by each female. In this experimental set-up, those of a twice-mated female’s eggs that hatch after the second mating will have been fertilized by her second mate, and hatching rate thus provides a direct measure of the competitive fertilization success of the second male (i.e., P2 [8]). We also counted the number of sterile eggs laid by each female in between her two matings, to use as a covariate in analyses of male competitive fertilization success.

### Effects of different fractions of accessory gland extract on mating receptivity

To characterize whether and how seminal fluid substances affect female receptivity to mating, we conducted a series of experiments where seminal compounds were injected into virgin females. Virgin females (0–2 days old) were injected with one of the size fractions of accessory gland extract (total volume, 0.1 μL; concentration, 0.03 mg/μL) and then kept individually in a Petri dish (height, 1.5 cm; diameter, 6.0 cm) with 1 mL of 20% sugar water in an Eppendorf tube as food. Each injected female was placed with a virgin male of 1–3 days of age in a glass tube, and we observed whether mating occurred or not within 60 min. Females were randomly assigned to either of four different groups, which were offered their single mating opportunity at either 3–5 h, 1, 2, or 3 days post injection (kept with food, as above). We also recorded the numbers of females that died during the experiment as well as the number of unfertilized eggs dumped prior to mating (females were virgin) within the Petri dish at each time point. These experiments were repeated in a second block identical to the first with the following two exceptions: (i) we used a different volume for the injections (0.05 μL; concentration, 0.05 mg/μL) and (ii) females were kept without food after injection.

### Effects of different fractions of accessory gland extract on female egg production rate

Virgin females (0–2 days old) were randomly assigned to one of the accessory gland size fractions injection treatments (total volume, 0.1 μL; concentration, 0.03 mg/μL). Injected females were then kept individually in a Petri dish (height, 1.5 cm; diameter, 6.0 cm) containing 10 mung beans which were replaced every 24h, and the number of eggs laid on the beans in each 24-h period was counted for 5 consecutive days, leaving us with five time-specific estimates of female egg production (0–1,1–2, 2–3, 3–4 and 4–5 post-injection). The females were then transferred to Petri dishes containing 50 mung beans and left there for life. To assess effects on lifespan, we recorded whether females were dead or alive once every 24-h. Because the immediate fecundity stimulating effects of male seminal fluid substances are known to differ from the long term effects in seed beetles [39], we restricted our inferential model to compare immediate effects (day 0–1) with long term effects (day 2–5). Only females that survived at least 5 days post-injection were included in the analyses.

### Statistical analyses

All statistical analyses were performed with JMP version 11.0.0 [47]. To evaluate the effects of male seminal fluid substances on the competitive fertilization success, we used generalized linear models of variance in the number of eggs hatched. These models used a binomial error distribution and a logit link function, and the total number of eggs laid by each female was used as the binomial denominator. The treatment (extract of either (i) accessory glands, (ii) testes, (iii) seminal vesicle and ejaculatory duct, (iv) control; or (i) high, (ii) medium, (iii) low molecular-weight fraction of accessory gland extract or (iv) control) and the length of the inter-mating interval (1 or 2 days) were used as factorial variables. The number of eggs laid in between the first and second mating was used as a continuous covariate, as this is known to affect P2 in this species [34]. All generalized linear models were fitted using Firth-adjusted maximum likelihood (for binomial responses; because of somewhat restricted sample sizes) and we compensated for overdispersion by appropriate scaling of deviances.

To evaluate the effects of size fractions of accessory gland extract on female mating receptivity, we used generalized linear logistic model of variance in remating with a binomial error distribution and a logit link function. The treatment, the number of days after injection (either 3–5 h, 1, 2, or 3 days post injection) and block (1 or 2) were used as factorial variables. The initial model included all main factors and all possible interactions, while our final inferential model included only those interactions that were significant (at α = 0.05). Female longevity was analyzed using an analogous model, with female being dead or alive at day 4 after injections used as the response. The number of eggs dumped by these females was analyzed in a generalized linear model with a Poisson error distribution and a log link function.

Female egg production (i.e., the number of eggs laid on mung beans) over time was analyzed in a repeated-measures ANOVA with treatment as the between-subjects factor and time as the within-subjects factor. Data were log transformed prior to fitting the model, to normalize the residual distribution. Total fecundity until female death was analyzed in a generalized linear model with a Poisson error distribution, and female life span was analyzed by using a Weibull model and a parametric survival test.

## Results

### Effects of male reproductive organ—derived extracts and fractions of accessory gland extract on sperm competitive success

**Fig 1:**
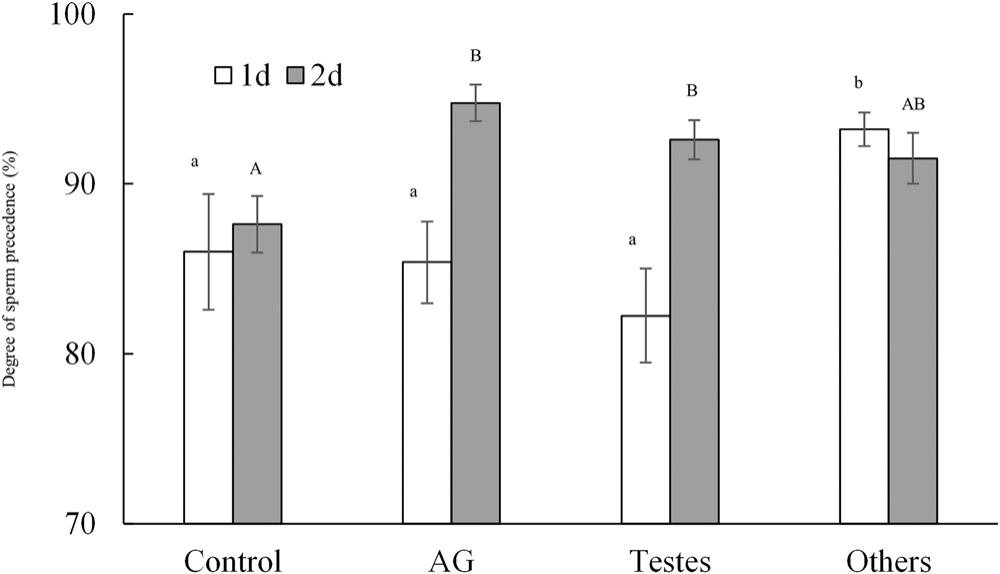
Competitive fertilization success of the second male to mate with females injected with extracts of male reproductive tracts. The figure shows the mean (± SE) degree of second male sperm precedence when females remated to a fertile focal male 1 or 2 days after first mating with a sterilized male. Females (N = 29–42 per day and treatment group) were injected with an aqueous solution of an extract of accessory glands (AG), testes, seminal vesicle and ejaculatory duct (others) or with Milli-Q water only as a control. Identical letters indicate no significant difference by sequential Bonferroni post-hoc tests [48] at α = 0.05.

Injection of extracts derived from different parts of the male reproductive tracts clearly affected male sperm competition success subsequent to the injections (Table 1), elevating the degree of sperm displacement compared to the control (Fig 1). Moreover, the effects of various extracts varied over time as indicated by the treatment × day interaction. The treatment showed significant effects both when re-mating occurred at day 1 (*χ* ^*2*^_*3*_ = 8.80, *P* = 0.032) and at day 2 (*χ* ^*2*^_*3*_ = 11.04, *P* = 0.012) after the first mating. However, while seminal vesicle and ejaculatory duct extracts had strongest effects in females re-mated at day 1, the accessory gland and testes extract showed strongest effects in females re-mated at day 2 (Fig 1).

**Table 1:** Generalized linear model of the effects of different male reproductive organ—derived extracts (treatment) and the number of days in between matings (days) on the sperm competitive success of the second male in double mating experiments (N = 29–42 females per day and treatment group).

Source	*df*	χ^2^	*P*
Treatment	3	12.80	0.005
Eggs between	1	0.48	0.490
Day	1	4.05	0.044
Treatment × Day	3	8.92	0.030

The number of eggs laid in between matings was used as a covariate.

**Fig 2:**
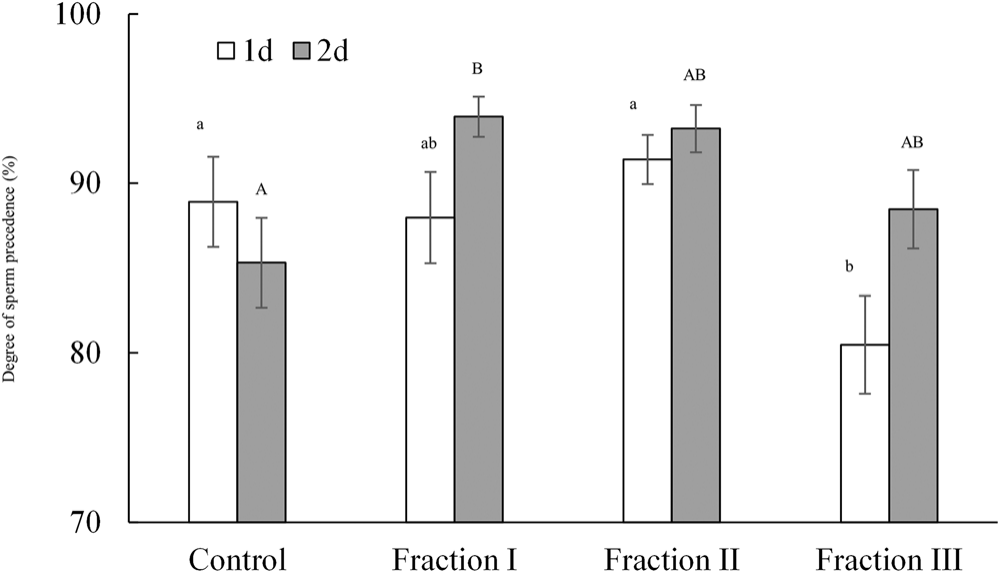
Competitive fertilization success of the second male to mate with females injected with extracts of male reproductive tracts. The figure shows the mean (± SE) degree of second male sperm precedence when females remated to a fertile focal male 1 or 2 days after first mating with a sterilized male. Females (N = 29–37 per day and treatment group) were injected with an aqueous solution of an extract of accessory glands (Fraction I [molecular weight: MW <25 kDa], Fraction II [MW, 25–50 kDa], or Fraction III [MW > 50 kDa]) or with Milli-Q water only as a control. Identical letters indicate no significant difference by sequential Bonferroni post-hoc tests [48] at α = 0.05.

Similarly, the various size fractions of male accessory gland extracts had a marked effect on sperm competition success and this effect varied depending on when females remated (Table 2). Again, our treatment showed significant effects both when re-mating occurred at day 1 (*χ* ^*2*^_*3*_ = 8.90, *P* = 0.031) and at day 2 (*χ* ^*2*^_*3*_ = 9.81, *P* = 0.020) after the first mating. While fraction III tended to decrease sperm displacement in females remated at day 1 after their first mating, fraction I increased sperm displacement in females remated at day 2 (Fig 2).

**Table 2:** Generalized linear model of the effects of three molecular-weight fractions of male accessory gland extract (treatment) and the number of days in between matings (days) on the sperm competitive success of the second male in double mating experiments (N = 29–37 females per day and treatment group).

Source	*df*	χ^2^	*P*
Treatment	3	13.85	0.003
Eggs between	1	10.55	0.001
Day	1	0.15	0.696
Treatment × Day	3	8.00	0.046

The number of eggs laid in between matings was used as a covariate.

### Effects of different fractions of accessory gland extract on female mating receptivity

**Fig 3:**
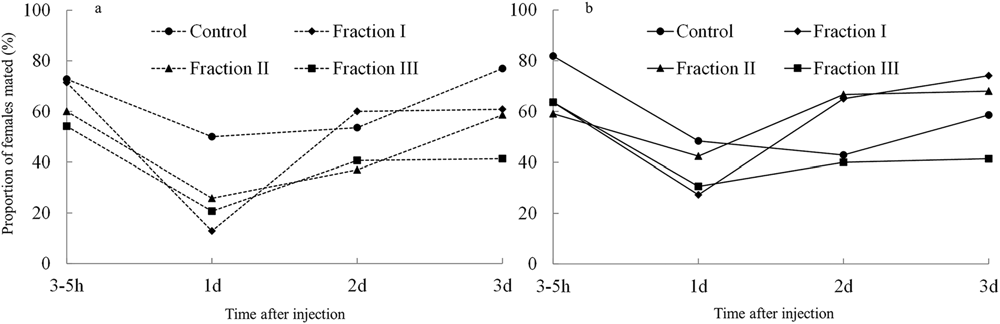
Receptivity to mating in virgin females (N = 20–36 per block and treatment group) injected with a fraction of male accessory gland extract (Fraction I [molecular weight <25 kDa], Fraction II [MW, 25–50 kDa], or Fraction III [MW > 50 kDa]) or with Milli-Q water only (control), at 3–5 h, 1, 2, and 3 d after injection. The two panels and represent the two experimental blocks.

Our treatment affected female mating rate, but the effects differed depending on the time that had passed since the injection (Table 3, Fig 3). We saw significant effects of our treatment only at 1 and 3 days after injection (3–5 h after injection: *χ* ^*2*^_*3*_ = 4.44, *P* = 0.218; 1 day after injection: *χ* ^*2*^_*3*_ = 14.71, *P* = 0.002; 2 days after injection: *χ* ^*2*^_*3*_ = 4.67, *P* = 0.198; 3 days after injection: *χ* ^*2*^_*3*_ = 10.93, *P* = 0.012). Furthermore, while treatment with fraction I or III of the accessory gland extract reduced female receptivity most compared to the control at day 1 day after injection, fraction III reduced female receptivity most at day 3 (Fig 3).

**Table 3:** Generalized linear model of the effects of different molecular-weight fractions of male accessory gland extract (treatment), the number of days after injection (days), and the experimental block (block) on female mating receptivity (N = 20–36 females per day, block and treatment group).

Source	*df*	χ^2^	*P*
Treatment	3	4.44	0.218
Day	3	60.32	< 0.001
Block	1	0.05	0.826
Treatment × Day	9	16.99	0.049
Treatment × Block	3	1.59	0.662
Day × Block	3	1.87	0.600
Treatment × Day × Block	9	6.49	0.690

**Fig 4:**
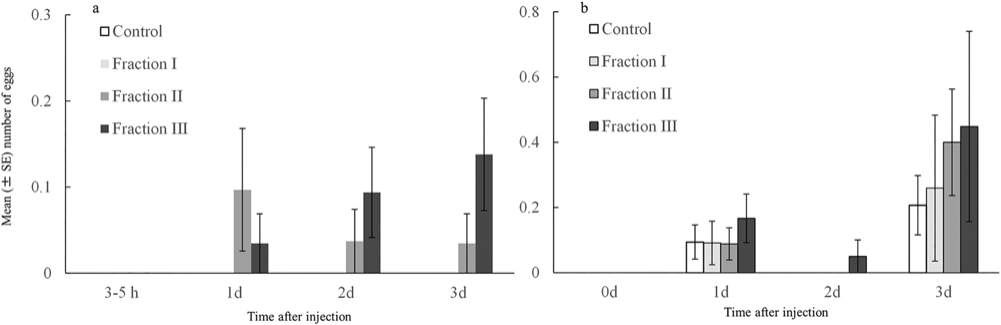
The total number of eggs dumped in the rearing vial by virgin females (N = 20–36 per block and treatment group) from the mating receptivity experiment, injected with a fraction of accessory gland extract (Fraction I [molecular weight <25 kDa], Fraction II [MW, 25–50 kDa], or Fraction III [MW > 50 kDa]), or with water only (control) at 3 to 5 h, 1, 2, and 3 d after injection. The two panels and represent the two experimental blocks.

Females injected with accessory gland extracts also dumped more eggs compared to the control, and the days after injection and the block also had significant effects on egg dumping (Table 4). Furthermore, females injected with fraction III dumped more eggs compared with females injected with either fraction I or control (Fig 4).

**Table 4:** Generalized linear model of the effects of different molecular-weight fractions of male accessory gland extract (treatment), the number of days after injection (days), and the experimental block (block) on the number of eggs dumped by females (N = 20–36 females per day, block and treatment group).

Source	*df*	χ^2^	*P*
Treatment	3	12.28	0.006
Day	3	51.89	< 0.001
Block	1	20.52	< 0.001

Non-significant interactions were removed to avoid over-parameterization.

Finally, the day at which females were offered to mate and block had significant effects on female the life span, while the injection treatment did not (Table 5). This suggests that the effects of injection with male derived proteins had at most marginal effects on female lifespan.

**Table 5:** Generalized linear model of the effects of injection of molecular-weight fractions of male accessory gland extract (treatment), the day at which females were offered a mating (days), and experimental block (block) on the number of dead females by day 3 after the injection of extracts (N = 20–36 females per day, block and treatment group).

Source	*df*	χ^2^	*P*
Treatment	3	7.65	0.054
Day	3	46.15	< 0.001
Block	1	18.54	< 0.001

Non-significant interactions were removed to avoid over-parameterization.

### Effect of different fractions of accessory gland extract on number of eggs laid

**Fig 5:**
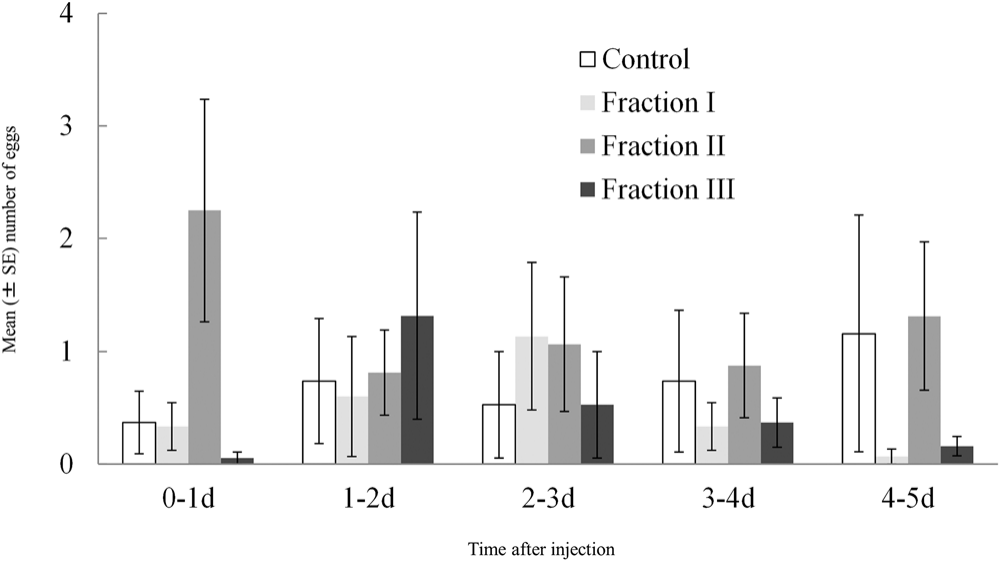
The number of eggs laid by virgin females (N = 16–19 per treatment group) provided with beans as oviposition substrate and injected with an aqueous solution of accessory gland extract (Fraction I [molecular weight <25 kDa], Fraction II [MW, 25–50 kDa], or Fraction III [MW > 50 kDa]), or Milli-Q water only (control), here shown separately for each of five consecutive 24-h periods.

Male accessory gland extracts had a significant effect on the egg production of virgin females and this effect varied with the time since injection (Table 6). This effect was primarily due to an immediate and strong oviposition-stimulating effect of fraction II during day 0–1, while treatment effects were less pronounced after this initial day (Fig 5). There was no significant difference among the three different size fractions in their effects on total number of eggs laid (*χ* ^*2*^_*3*_ = 1.84, *P* = 0.606) or on female life span (*χ* ^*2*^_*3*_ = 2.100, *P* = 0.552).

**Table 6:** Repeated measures ANOVA of the number of eggs laid by virgin females following injections with no, a high, medium, or low molecular-weight fraction of male accessory gland extracts (treatment) (N = 16–19 females per treatment group).

Souce	SS	*df*	*F*	*P*
Between-subjects				
Treatment	0.411	3	4.16	0.009
Error	2.108	64		
Within-subjects				
Time	7.859	1	302.26	<0.001
Time × Treatment	0.345	3	4.42	0.007
Error	1.664	64		

## Discussion

It is often stated that male seminal fluid proteins aid in male sperm displacement in animals with internal fertilization. However, to our knowledge, previous demonstrations of this are limited to *D*. *melanogaster* [23,24,49,50]. We found that, in particular, the male accessory reproductive glands of *C*. *maculatus* contain substances that elevate the competitive fertilization success of second males, but that additional substances in the testes also had similar effects. One or more relatively short proteins (≥25 kDa) are primarily responsible for this elevation. Ongoing proteomic work in our group on the accessory glands of *C*. *maculatus* has revealed a large number (more than 100) of accessory gland proteins (henceforth, acps) that are transferred to female at mating, ranging in size from less than 10 kDa to almost 100 kDa. These acps are, however, not yet identified. However, our work confirms that male derived seminal fluid substances indeed determine the relative competitive fertilization success of males in sperm competition.

In *D*. *melanogaster*, Acp36DE elevates both the first and second male sperm competitive ability by increasing the rate of sperm storage in the female spermatheca [19,20]. Furthermore, a comparative analysis of gene expression, transcript abundance and phenotypic differences showed that Acp33A was associated with both first and second male sperm competitive ability, and that another acp (CG17331) correlated with second male sperm competitive ability [22]. Interestingly, Acp62F is known to reduce first male sperm competitive ability but has no sizeable effect on second male sperm competitive ability [24]. In *C*. *maculatus*, although we found that one or more low molecular-weight fraction acps enhanced the sperm competitive ability of second males that mated 2 days after a female’s first mating, one or more high molecular-weight fraction acps instead reduced the sperm competitive ability of second males that mated 1 day after a female’s first mating (Fig 2). This suggests that large proteins (>50 kDa) act to increase sperm defensive function of the first male to mate (i.e., P1) in *C*. *maculatus*. One might predict, therefore, that males should tailor their ejaculate according to mating order, such that the relative amount of acps with role-specific function is tuned to female mating status. Such effects have been observed in other insects [10,51]. In fact, ejaculate composition is known to be affected by various factors in a wide variety of taxa [52], including seed beetles [53]. However, it is also possible that some acps may have negative pleiotropic side-effects. For example, acps that stimulate oviposition may interfere with sperm uptake to the spermatheca as eggs pass down the oviduct. In concordance with this possibility, our results showed that large acps both stimulated egg dumping and egg laying, as well as reduced second male competitive fertilization success.

Our results also show that seminal fluid substances have a range of other effects in females, and these results are well aligned with previous studies of the effects of seminal fluid substances in insects in general [15] and in seed beetles in particular [38,39]. For example, in *D*. *melanogaster*, a peptide synthesized in the ejaculatory duct (Dup99B) reduces female mating receptivity but increases egg production and egg laying [25] and ovulation-stimulating substances are synthesized in the accessory glands and ejaculatory duct in *D*. *biarmipes* [26]. The accessory glands and testes of male yellow fever mosquito (*Aedes aegypti*) and common house mosquito (*Culex pipiens*) both produce oviposition-stimulating substances [27] and those of the rice leaf bug *Trigonotylus caelestialium* produce a mating receptivity substance [28]. Here, we found that male *C*. *maculatus* reproductive organ—derived extracts both reduced receptivity to mating in females and stimulated egg laying. Size fraction I temporarily reduced mating receptivity at 1 day after injection, whereas fraction III had a longer lasting effect and reduced mating receptivity at both 1 and 3 days after injection (Fig 3). Further, fraction III and fraction II both stimulated egg laying on larval host beans (Fig 5) and fraction III also stimulated egg dumping (Fig 4).

Male seminal substances normally confer benefits to males but the receipt of some acps can be costly for females [54]. For example, in *D*. *melanogaster*, a sex peptide (Acp70A) that stimulates egg laying and reduces female receptivity can also decrease female lifespan and lifetime offspring production under some environmental conditions [55–57] and Acp62F seem to have toxic systemic side-effects in females [58]. Toxic effects of seminal fluid proteins in females have also been reported in two other seed beetles: *Acanthoscelides obtectus* [59] and *C*. *chinensis* [39]. In the present study, we found no significant net effects of male reproductive organ—derived extracts on female longevity. Considering the fact that more than 100 distinct acps are transferred to females at mating in *C*. *maculatus*, it remains a possibility that different acps may differ in the sign of their effect on female longevity such that the net effect is insignificant.

Hotzy et al. [37] suggested that male genital spines in *C*. *maculatus* may, at least in part, function to pierce the wall of the female copulatory duct, thus allowing a more rapid and/or efficient passage of acps into the female haemolymph. This suggestion was based on the observations that (i) males with long spines have higher competitive fertilization success, (ii) males with long spines cause more internal injury to females [37] and (iii) male seminal fluid substances disperse into the female body more rapidly after having mated to males with long spines. Our current work provides support for this hypothesis, to the extent that we present evidence for a causal link between the presence of male seminal fluid substances in the female haemolymph and elevated male competitive fertilization success.

Our results also revealed that several effects of male derived substances were to some extent context dependent. First, the effects on sperm displacement rates differed depending on female inter-mating interval. For example, accessory gland extract elevated the competitive fertilization success of second males 2 days after the first mating but this effect was not seen 1 day after the first mating. Second, male substances reduced mating receptivity in females 1 day after injections but not 2 days after injections. These results predict that males might show ejaculate tailoring [7,10], modifying the composition of the ejaculate according to female mating status, the time since the female last mated and possibly female age and condition. It remains to be explored, however, whether males are capable of such adaptive fine-tuning of ejaculate composition.
